# Measurement of Cervical Regression and Optimizing Brachytherapy Schedule Concurrently with External Beam Radiation Therapy in Cervical Carcinoma

**DOI:** 10.7759/cureus.5316

**Published:** 2019-08-03

**Authors:** Parthasarathy Vedasoundaram, Santhosh Vandanasetti, Kannan Periasamy, Saravanan Kandasamy

**Affiliations:** 1 Radiation Oncology, Jawaharlal Institute of Postgraduate Medical Education and Research, Puducherry, IND; 2 Radiotherapy and Oncology, Postgraduate Institute of Medical Education and Research, Chandigarh, IND; 3 Medical Physics, Jawaharlal Institute of Postgraduate Medical Education and Research, Puducherry, IND

**Keywords:** carcinoma cervix, cervical cancer, cervical regression, external beam radiation therapy, brachytherapy

## Abstract

Introduction

This study aimed to measure cervical regression during external beam radiation therapy (EBRT) and optimize the scheduling of brachytherapy concurrently with EBRT.

Methods

Fifty consecutive patients with carcinoma of the cervix stage IIA to IIIB received concurrent chemoradiotherapy with weekly Cisplatin 40 mg/m^2^. Cervical regression was evaluated using serial CT scans obtained before and during concurrent chemoradiotherapy (on the third, fourth, and fifth weeks). High dose rate brachytherapy was introduced after 30Gy of EBRT. A total of 25.5Gy in three fractions were delivered during the third, fourth, and fifth weeks of EBRT. Cervical volumes were recorded from the CT scan for cervical regression.

Results

The mean cervical volume at baseline (i.e., before the start of treatment) was 85.53 cubic centimetres (cc). The mean cervical volumes at the end of the third, fourth, and fifth week were 28.95cc, 24.92cc, and 21.80cc, respectively. The mean cervical regression from baseline to the end of the third, fourth, and fifth week was 60%, 65%, and 69%, respectively. The time for 50% cervical regression was calculated to be 18 days and occurred around 27Gy of EBRT.

Conclusion

More than 50% of cervical regressions occur at the end of the third week (i.e., after delivery of 30Gy of EBRT), so it is optimal to introduce brachytherapy at the end of the third week. A conventional point-based plan can cover the high-risk clinical target volume (HRCTV) if the volume is <25cc, but an HRCTV >25cc may be well covered with optimization or a combination of intracavitary and interstitial brachytherapy.

## Introduction

Cancer of the cervix is the second most common cancer reported in the Indian female population [1]. Most cases are locally advanced, with nearly 70% in Fédération Internationale de Gynécologie et d’Obstétrique (FIGO) Stage IIB or Stage III. Curative radiation therapy in carcinoma of the cervix is usually a combination of external beam radiation therapy (EBRT) and brachytherapy. Over the years, the external beam radiotherapy for carcinoma of the cervix has been well standardized and uniform; however, the brachytherapy component carries tremendous variation. Often, brachytherapy is used as a boost after EBRT for locally advanced disease as it delivers a high radiation dose to the tumor and high-risk clinical target volume (HRCTV) while optimally sparing the surrounding normal tissue.

Various studies have shown that pelvic control and survival rate is compromised when treatment time lasts beyond eight weeks [2-5]. Perez et al. observed that if treatment time was prolonged beyond eight weeks, the failure rate was about 0.85% per day when the total dose delivered was ≥85Gy to point A [2]. The available literature on the involution of the cervix during chemoradiotherapy is very sparse. Lee et al. showed that chemo-radiation treatment of cervical cancer would rapidly involute the cervix [6]. The time taken for 50% tumor regression was 21 days and occurred after 30.8Gy.

These facts kindled interest in measuring cervical regression with the intent to optimize the use of brachytherapy insertions concurrently with EBRT. The total duration of treatment can be shortened to fewer than six weeks, thus, affording a chance for better local control. We used CT scans for contouring the HRCTV and measuring cervical regression, given that a high-volume center place makes getting a slot for MRI next to impossible. Also, standard Viswanathan et al. guidelines are available for HRCTV contouring based on CT images, which made CT-based contouring to measure cervical regression a logical choice [7].

## Materials and methods

**Figure 1:**
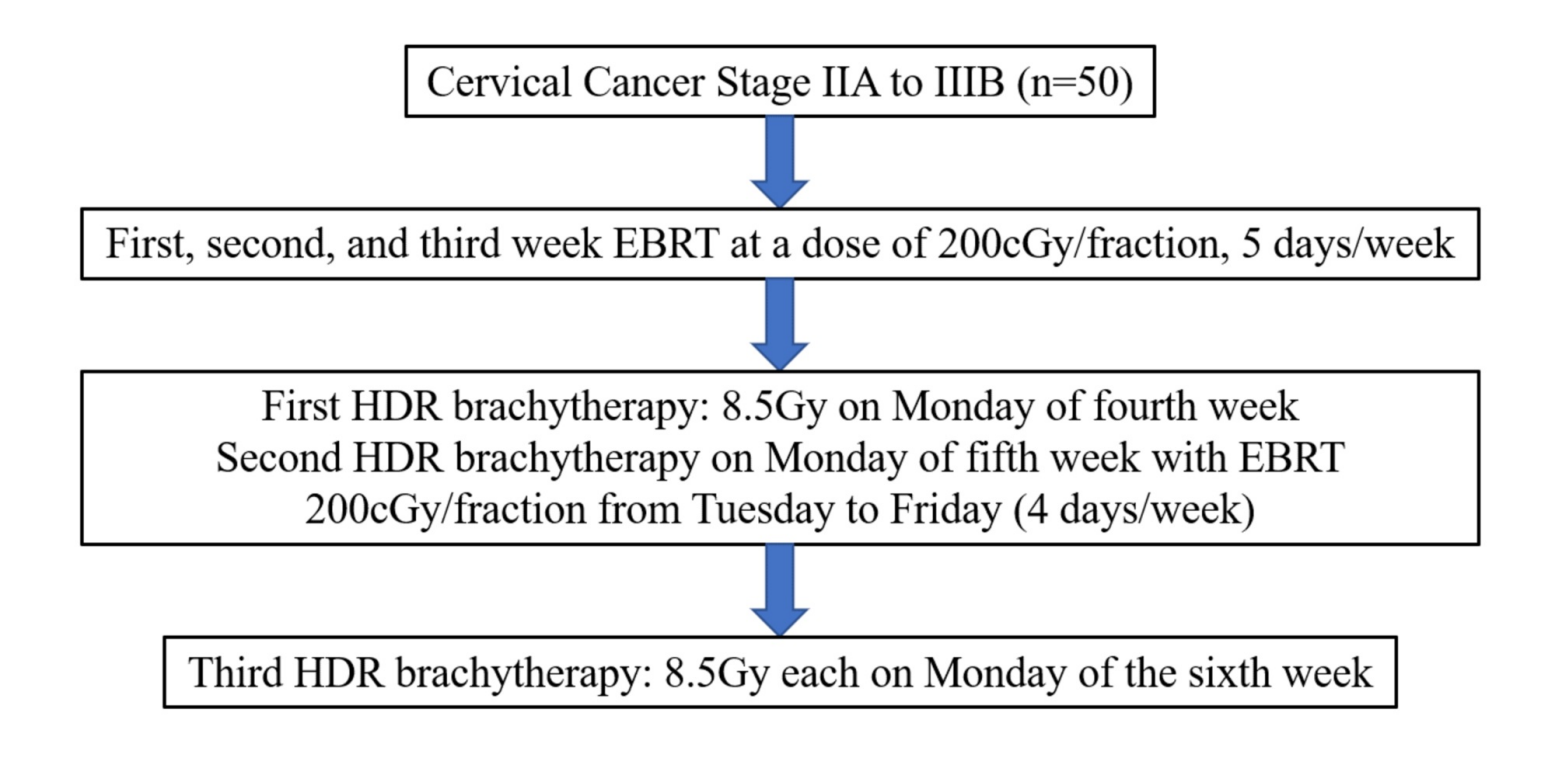
Study design EBRT-External Beam Radiation Therapy HDR-High Dose Rate Gy-Gray

Fifty consecutive patients with squamous cell carcinoma of the cervix in Stage IIA to IIIB (without the involvement of the lower third of the vagina) with healthy renal, hepatic parameters and Eastern Cooperative Oncology Group (ECOG) performance score of zero to two were included in the study. Patients with Stages IIIA, IVA, and IVB and patients unfit for high dose rate (HDR) brachytherapy after receiving 30Gy of EBRT were excluded and allowed to continue EBRT for 46Gy with midline shielding up to 50Gy. Brachytherapy was considered as per regular institution protocol (i.e., 8.5Gy to Point A x three fractions with one week between each fraction). The study patients received concurrent chemoradiotherapy with weekly cisplatin 40 mg/m2 up to a maximum of five cycles. EBRT was delivered by four-field box technique with corner shielding by multileaf collimator. A total dose of 50Gy/25 fractions with 2Gy per fraction per day was delivered by EBRT with midline shield after 46Gy of EBRT. Brachytherapy was delivered by three fractions of HDR intracavitary application (8.5Gy to Point A x three fractions). The first fraction of HDR brachytherapy was introduced after 30Gy of EBRT; the second and third applications were done seven days after the previous application. The study design is depicted in Figure 1.

Brachytherapy procedure

We used standard compatible applicators for an HDR Ir 192 brachytherapy machine, either Tandem and Ring or Tandem and Ovoid applicator depending on the depth of the fornices were used. The brachytherapy procedure was done as per standard institution protocol with patients under spinal anesthesia. A post-implant simulation CT scan was done; the volumes of organs-at-risk were visualized, and the bladder and rectum were delineated on each CT slice. Treatment planning was done using Brachyvision version 10 (Varian Medical Systems, Palo Alto, CA). The dose-volume histograms generated by the planning system were used for recording the volume doses for all relevant organs. Point doses were calculated using the International Commission of Radiation Units and Measurements reference points. We used the CT standardized contouring guidelines by Viswanathan et al. [7].

Measurement of cervical regression

Patients were evaluated during concurrent chemoradiotherapy for assessment of cervical regression via serial CT scans obtained in the treatment position before and during the third, fourth, and fifth week of treatment. HRCTV was contoured on each CT scan slice, and volume was calculated and recorded in cubic centimeters (cc) using Eclipse software version 10 (Varian Medical Systems, USA). The serial volumes were analyzed for cervical regression, and V150, V200, D90, and D100 of the HRCTV were computed.

## Results

Patient characteristics

The patients’ characteristics are shown in Table 1. Fifty consecutive patients with Stage IIA to IIIB carcinoma of the uterine cervix treated with chemo-radiation formed the study group. The median age of the patient was 45 years (range, 30 to 60 years), and the mean age was 46 years (SD, 8.2). The mean tumor size was 84.5cc with a range of 28 to 221cc. Thirty-two percent of patients had a pretreatment ECOG performance status (PS) of 0, and 68% belonged to PS 1. Forty-four percent had Stage IIB, 32% had Stage IIIB, and 24% had Stage IIA disease at presentation. Most patients (84%) received four or more cycles of cisplatin, while 16% received three cycles of chemotherapy.

**Table 1:** Patient characteristics ECOG PS, Eastern Cooperative Oncology Group performance status.

Patient characteristics (n=50)	
Age	
Median (years)	45
Range (years)	35-60
Tumor size	
Mean (cm^3^)	84.5
Range (cm^3^)	28-221
ECOG PS	% of patients
0	32%
1	68%
Stage	% of patients
IIA	24
IIB	44
IIIB	32
No. of cycles of Cisplatin	% of patients
3	16
4	40
5	44

Cervical regression analysis

The mean cervical volume at baseline (i.e., before starting treatment) was 85.53cc. The mean cervical volume at the end of the third, fourth, and fifth week was 28.95cc, 24.92cc and 21.80cc, respectively. The mean cervical regression from baseline to the end of the third week was 60%. The mean cervical regression from baseline to the end of the fourth and fifth weeks were 65% and 69%, respectively, as shown in Table 2.

**Table 2:** Cervical volume at different weeks CC - Cubic Centimetres SD- Standard Deviation CI - Confidence Interval

Tumor volume	Mean (cc)	SD	95% CI of the difference	
			Lower	Upper
Base line	84.53	49.27	64.19	104.87
End of third week	28.95	10.64	24.55	33.34
End of fourth week	24.92	9.01	21.20	28.64
End of fifth week	21.80	7.61	18.65	24.94

**Figure 2:**
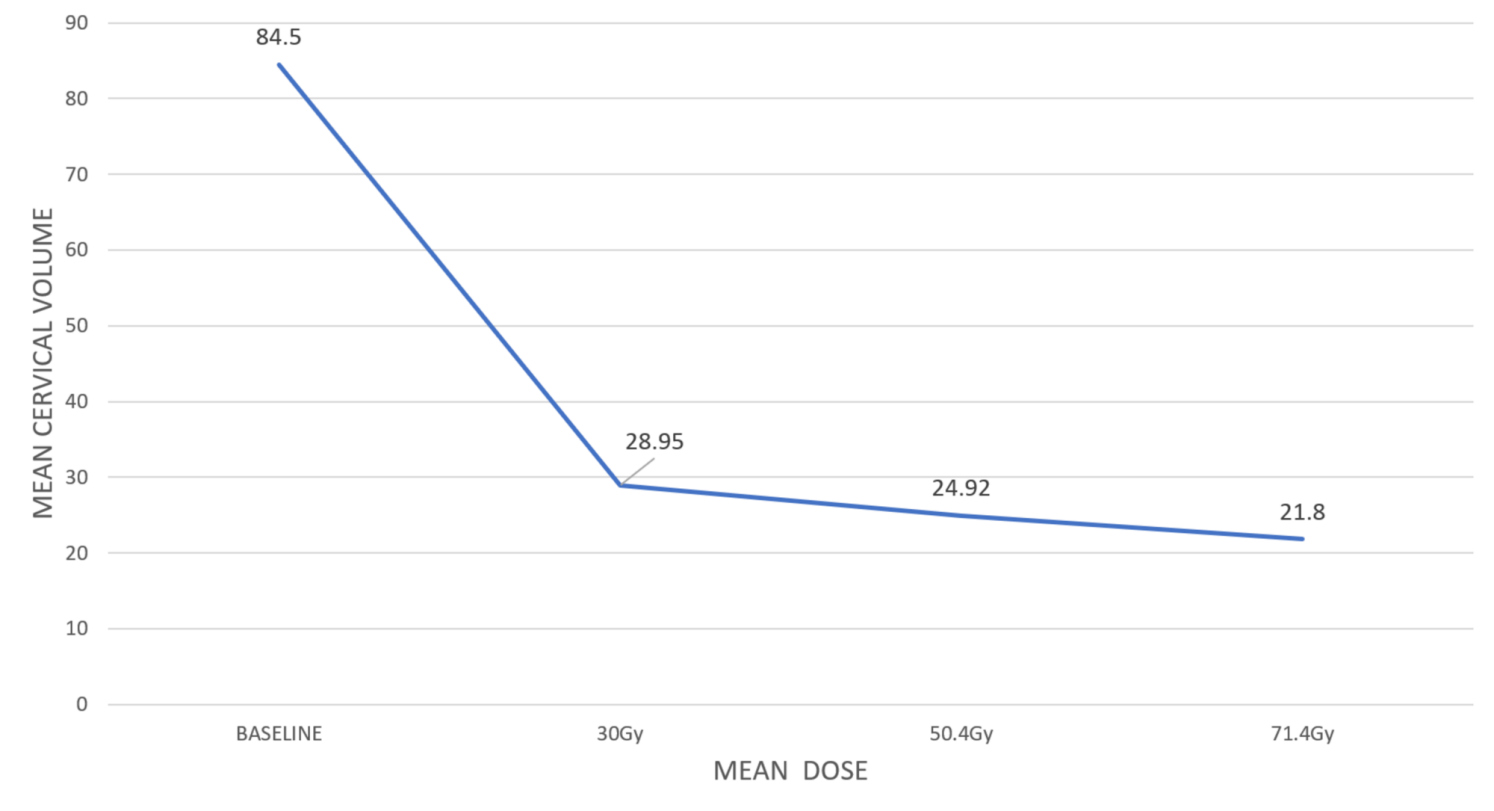
Mean cervical volume as a function of mean dose

**Figure 3:**
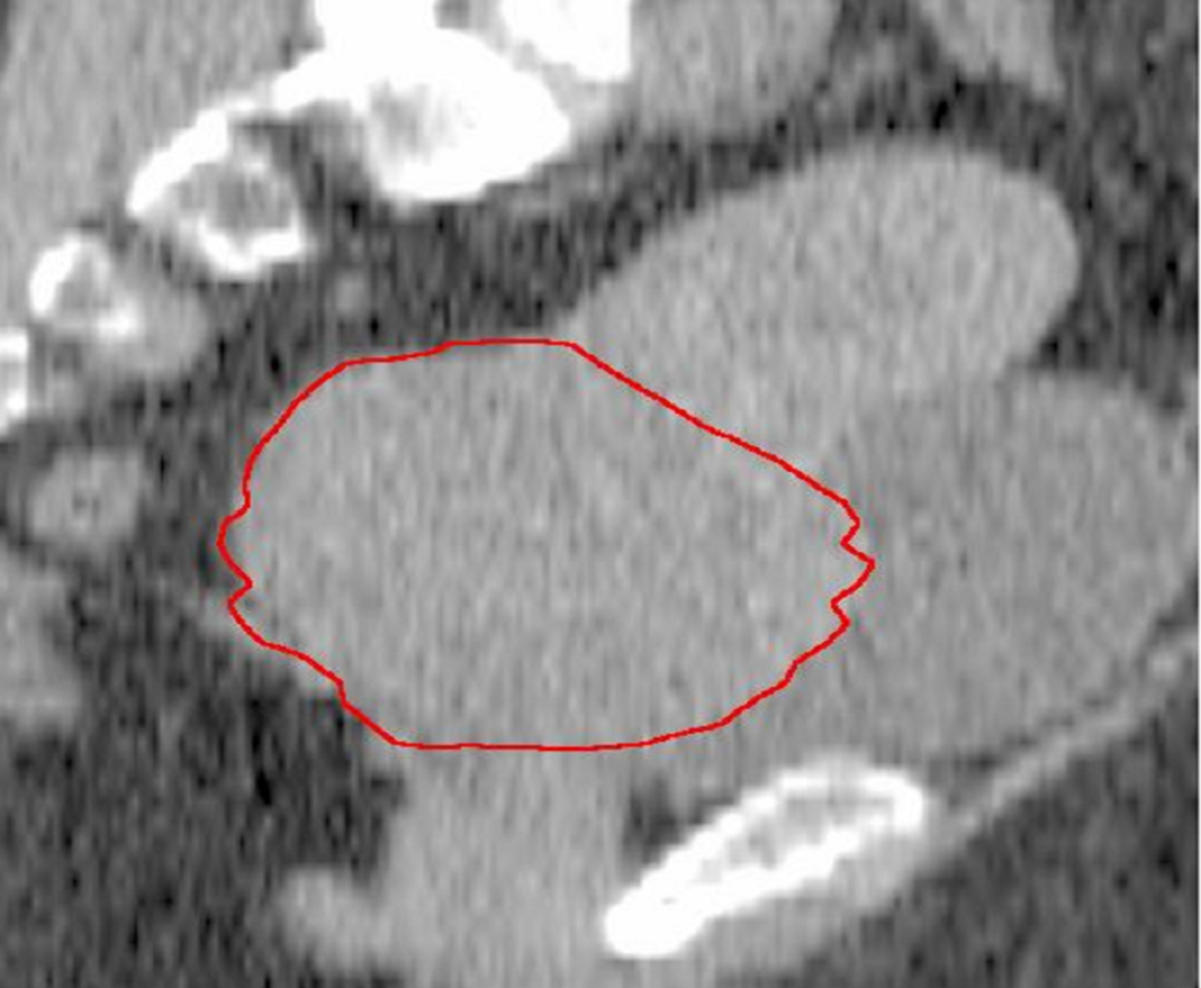
Cervical volume before the start of treatment

**Figure 4:**
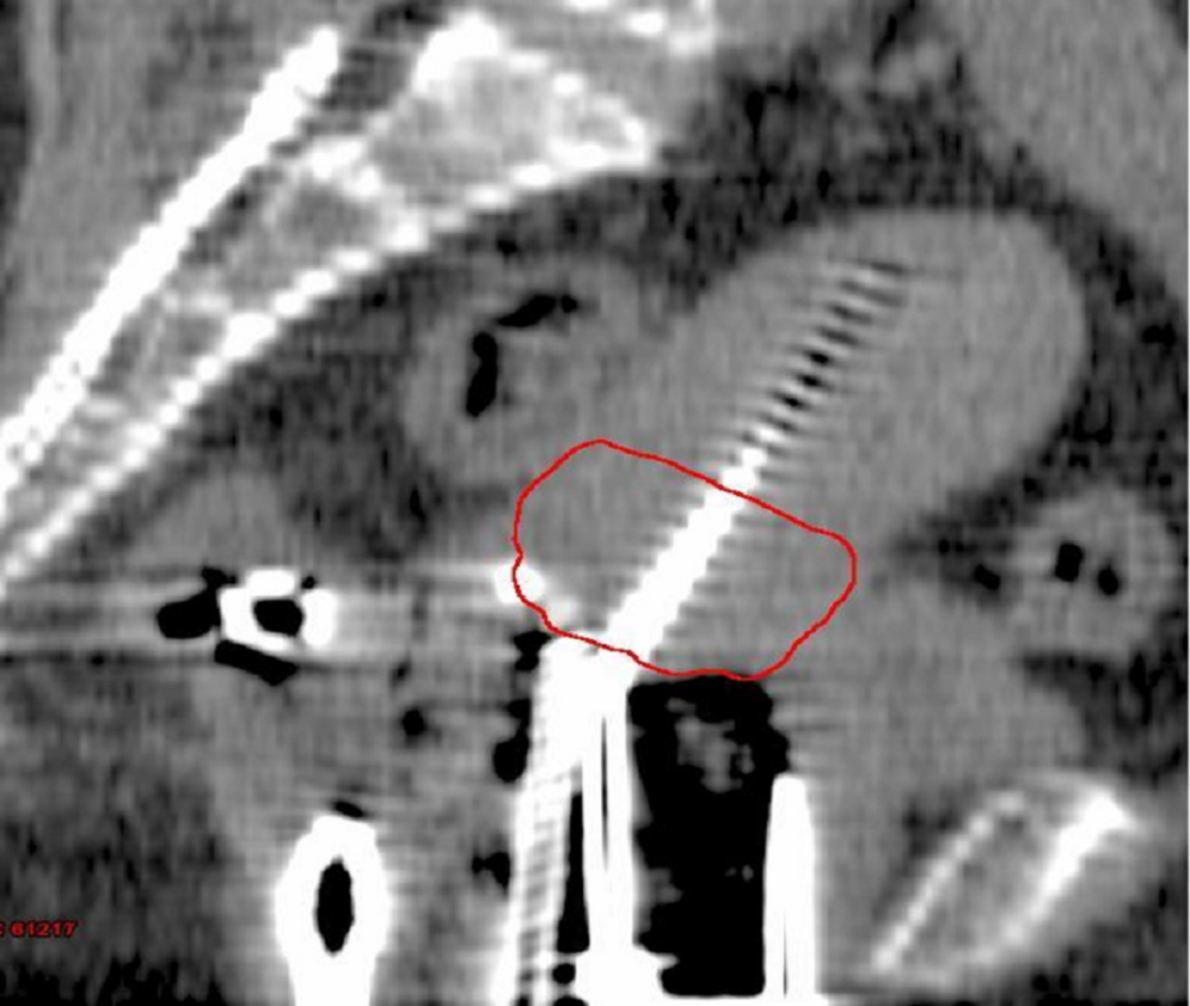
Cervical volume at the end of the third week of the same patient

The mean cervical volume was different between all four measurements as depicted in Figure 2 (F=43.0, P<.0001). Post hoc tests using the Bonferroni correction revealed a significant reduction between the baseline measurement and measurement at the third week (84.53 vs.28.95, P<.0001). At 30Gy, mean cervical volume was 28.9cc; it was decreased by 60% from baseline. The absolute decrease in mean cervical volume at 30Gy was 55.5cc. The time for 50% cervical regression was 18 days and occurred around 27Gy of EBRT (per logistic regression analysis). There was a strong positive correlation between initial cervical volume and percentage of cervical regression at 30Gy (r=0.744, P<.0001). The cervical volumes of a patient before the start of treatment and at the end of the third week of treatment are shown in Figures 3, 4.

Target volume parameters

The mean HRCTV was 25.22cc. (SD, 9.4). The mean dose to 90% of HRCTV (D90) was 8.41Gy (98.94% of the point A dose). Interfraction variations in mean doses to HRCTV are shown in Table 3.

**Table 3:** Mean doses to HRCTV at each brachytherapy HRCTV- High Risk Clinical Target Volume HDR- High Dose Rate

		HRCTV	D90 (%)	D100 (%)	V100 (%)	V150 (%)	V200 (%)
First HDR brachytherapy	Mean	28.95	95.24	62.3	83.44	54.82	33.97
	SD	10.5	22.1	17.88	14.1	14.7	9.96
Second HDR brachytherapy	Mean	24.92	97.74	63.9	85.64	57.00	35.92
	SD	8.92	22.8	18.42	11.44	13.80	10.98
Third HDR brachytherapy	Mean	21.80	103.85	70.1	90.3	61.96	39.29
	SD	7.54	27.6	20.6	12.0	15.5	12.1

**Figure 5:**
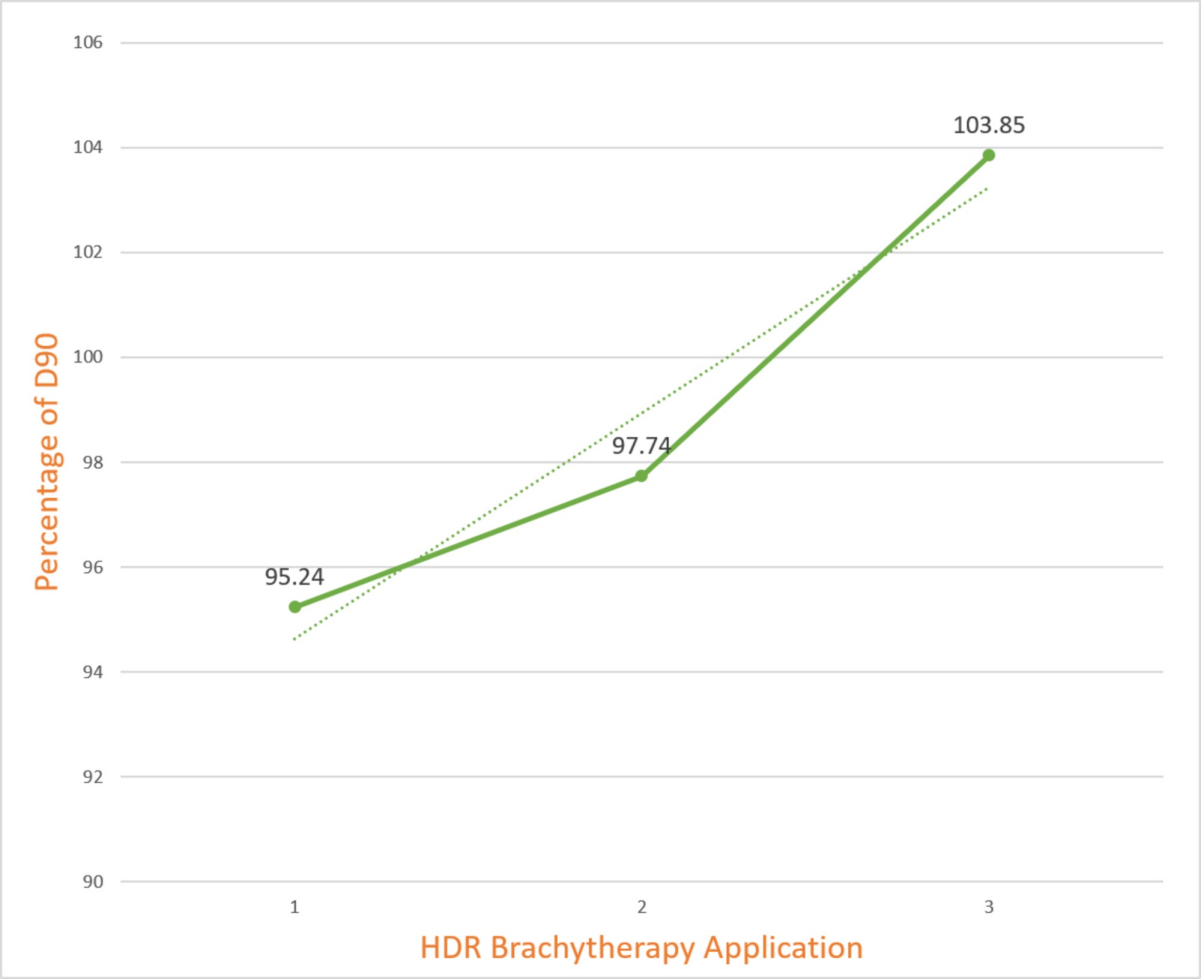
Interfraction variation of D90 of HRCTV HDR-High Dose Rate HRCTV-High Risk Clinical Target Volume

The interfraction variation of D90 of HRCTV is shown in Figure 5. The mean D90 value differed statistically significantly between the three brachytherapy applications (F=3.536, P<0.05). The difference in mean HRCTV between the applications with D90 ≥100% and those with D90 ≤100%was statistically significant (p=0.0001). The applications with D90 ≤100% had significantly higher HRCTV (30.44±9.5) compared to D90 ≥100(19.25±4.7).

Logistic regression was performed to ascertain the effect of HRCTV on the plan acceptability (if a plan with D90≥100 is acceptable). The logistic regression model was statistically significant (chi-square value, 85.49; p<.0001). For every unit increase in the HRCTV, the odds of the treatment plan getting rejected (i.e., requiring optimization) was 1.34. Decreasing HRCTV was associated with an increased likelihood of plan having D90≥100. The cutoff for HRCTV to increase the plan acceptability (D90≥100) was found to be 25cc. Plans with HRCTV ≥25cc had significantly lower D90 values (79.7±14%) compared to plans with HRCTV ≤25cc (111±21%).

## Discussion

The radiation treatment for cancer of the cervix consists of both EBRT and brachytherapy. Multiple studies have shown that treatment time prolongation greater than eight weeks resulted in increased local failures and decreased overall survival. This study aimed to measure cervical regression and introduce the brachytherapy schedule concurrently with EBRT with the intent of decreasing the overall treatment to fewer than six weeks.

Cervical regression

The data from the study indicate that 50% cervical regression occurred at 27Gy at about 18 days, and the mean cervical regression at the end of the third week was 60% with a decrease of 55.55cc in absolute volume, which is similar to the observation made by Lee et al., where they found 50% cervical regression at a median dose of 30.6Gy at about 21 days [6]. Another study on cervical regression by Van De Bunt et al.found that mean cervical regression at the third week or at 30Gy of EBRT was 46% [8].

The baseline cervical volume was 84cc, and at the end of 45Gy, mean cervical volume reduction was 65% in our study. Unlike the observations of Van De Bunt et al., a strong positive correlation was identified between initial cervical volume and percentage of cervical regression which was statistically significant (r=0.744; P<.0001) [8]. These findings pave the way for interdigitating brachytherapy during EBRT; at the end of the third week (i.e., the completion of 30Gy) may be the most optimal time for the first brachytherapy application.

Target volume coverage

A total of 150 brachytherapy applications were conducted in the study. D100 and D90 are the parameters to be analysed for HRCTV [9-11]. D100 changes with contour irregularities; therefore, it is not a sensitive indicator. However, D90 does not change with contour irregularities, making it the optimal parameter for study. The mean HRCTV D90 in the study was 8.41Gy (SD, 2.05). This was 0.09Gy less than the prescribed point A dose (8.5Gy). In 70 applications (46%), the D90 of the HRCTV was lower than the prescribed dose. The difference in mean HRCTV between the applications with D90 ≥100% and those with D90 ≤100% was 11.19cc. The applications with D90 ≤100% had significantly higher HRCTV (30.44±9.5) compared to D90 ≥100 (19.25±4.7). Logistic regression showed the cutoff for HRCTV to improve plan acceptability (D90 ≥100) was 25cc. The plans with HRCTV ≥25cc had a D90 value of 79.7% (SD, 14) whereas plans with HRCTV ≤25cc had a D90 value of 111% (SD, 21). This shows that there is a negative correlation between HRCTV and the D90 of HRCTV. As the HRCTV increases, point-based prescription may not cover the HRCTV adequately.

Tanderup et al. observed that plans with HR-CTV <31cc was well covered by point-based prescription in 94% of patients [12]. Optimization improved the HRCTV D90 in plans with HRCTV >31cc compared with point-based plans (72% vs. 25%).

**Figure 6:**
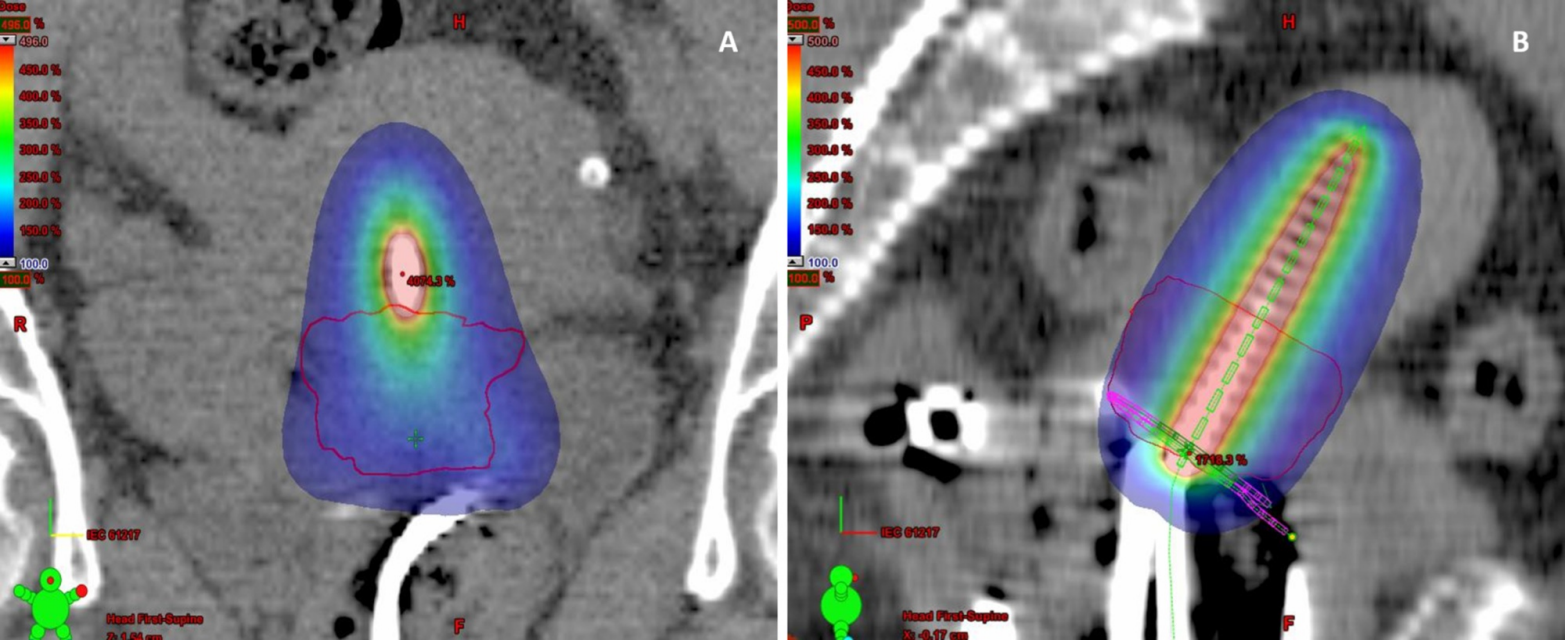
CT image (A, B) showing coverage of HRCTV (<25cc) by 100% isodose color wash HRCTV-High Risk Clinical Target Volume

**Figure 7:**
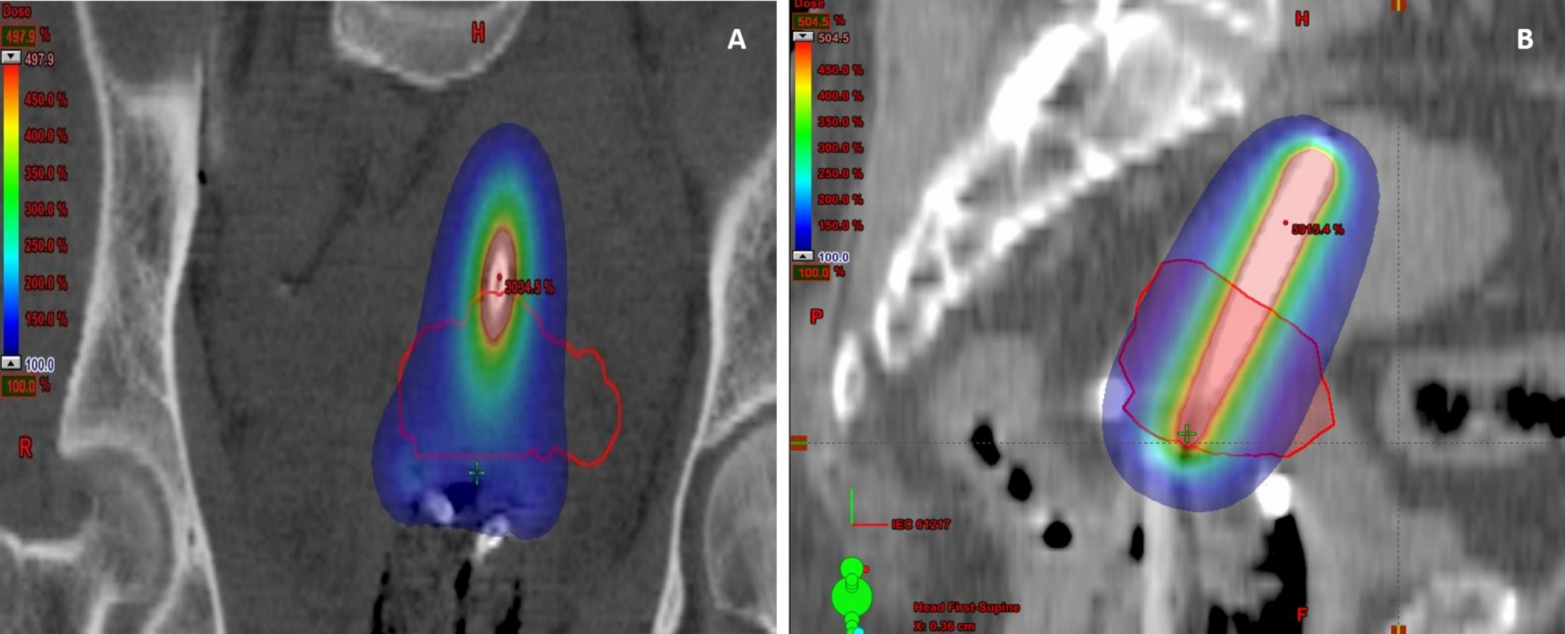
CT image (A, B) showing inadequate coverage of HRCTV (>25cc) by 100% isodose color wash HRCTV-High Risk Clinical Target Volume

Conventional Point A prescription was able to cover the HRCTV in most of the applications if the HRCTV <25cc. However, when HRCTV >25cc, conventional point A prescription would not be adequate to cover the HRCTV as shown in Figures 6, 7. The possible treatment options to improve dose coverage while sparing of healthy tissues are to continue EBRT up to 45 to 50Gy or until tumor regression occurs <25cc, but the duration of overall treatment cannot be reduced to fewer than six weeks. Thus, other options are found in image-based optimization or combined intracavitary-interstitial brachytherapy.

 

## Conclusions

Optimizing brachytherapy schedule concurrently with EBRT is feasible in carcinoma of the cervix with locally advanced disease. More than 50% cervical regression occurs at the end of the third week (i.e., after delivery of 30Gy of EBRT), so it is optimal to introduce brachytherapy at the end of the third week. It is possible to decrease the overall treatment by introducing brachytherapy at the end of the third week. A conventional point-based plan can cover the HRCTV if the volume is less than 25cc, but HRCTV greater than 25cc may be well covered with a combination of intracavitary plus interstitial brachytherapy. Further research in locally advanced cervical cancer is warranted to incorporate interstitial brachytherapy with intracavitary applications, especially for bulky disease, particularly for patients with parametrial and vaginal involvement.
